# Risk Assessment of Harmful Algal Blooms in Salmon Farming: Scotland as a Case Study

**DOI:** 10.3390/toxins17010035

**Published:** 2025-01-13

**Authors:** Fatima Gianella, Michael T. Burrows, Keith Davidson

**Affiliations:** Scottish Association for Marine Science—UHI, Oban PA37 1QA, UK; mf.gianella@gmail.com (F.G.); michael.burrows@sams.ac.uk (M.T.B.)

**Keywords:** HABs, fish-killing algae, risk assessment, *Karenia mikimotoi*, Scotland, salmon farming

## Abstract

This study explored harmful algal bloom (HAB) risk as a function of exposure, hazard and vulnerability, using Scotland as a case study. Exposure was defined as the fish biomass estimated to be lost from a bloom event, based on the total recorded annual production. Hazard was estimated from literature-reported bloom events. Vulnerability was calculated from records of the number of employees (2020), as an estimate of aquaculture-based employment. The dinoflagellate *Karenia mikimotoi* was identified as the HAB species with the highest frequency of reported bloom events in Scotland, with variable spatial and temporal reports, but environmental and climatological variables regulating these events are currently unknown. The Shetland Islands region exhibited the highest combined HAB risk, with the highest scores in all three components. Vulnerability was particularly important to overall risk within an island setting, where a larger proportion of the population was dependent on aquaculture. The analysis demonstrated the potential to evaluate the economic and social consequences of HAB events on the aquaculture industry. As fish-killing HABs and fish health impacts are likely under-reported, more transparent reporting of events and related fish health and physiological consequences is recommended for a more quantitative application of this approach.

## 1. Introduction

Salmon (*Salmo salar*) farming has steadily increased globally in volume over time, with this species currently ranked sixth of the most farmed fish worldwide, reaching 2436 thousand tonnes in 2018 [1]. The five countries with the highest farmed salmon production (comprising 90% of the worldwide total) are Norway (55.88% tonnes of live weight in 2022), Chile (27.33%), the United Kingdom (6.09%), the Faroe Islands (3.91%) and Canada (3.89%) [2]. In all cases, the industry is significant in national economic terms, with, for example, salmon being Scotland’s largest food export by volume and value. This activity is mainly carried out in enclosed fjords or loch environments generally located in rural coastal areas, where it can be a major local source of employment. Fjords are typically sheltered and prone to calm conditions that can induce stratification, which in turn can stimulate phytoplankton growth and hence can often provide the environmental conditions to support the development of harmful algal blooms (HABs), potentially leading to the mortality of farmed fish [3]. These events have had severe socio-economic impacts, with a global annual loss estimated at US$ 8 billion [4]. Particular extraordinary events have caused ~US$ 800 million in economic losses in Chile in 2016 [5] and US$ 82 million in Norway in 2019 [6]. The quantification of different components of HAB risk is therefore required to better support industry and local government in the production of strategies to decrease or minimise HAB impacts and enhance regional sustainable development.

The study of HAB events as a hazard to salmon farming activities is crucial to the development of risk assessments capable of providing an enhanced understanding of social and economic impacts in a particular location [7]. This information is useful for stakeholders such as policymakers, industry sectors (aquaculture, insurance) and those with livelihoods depending on fish farming. Risk can be estimated as a function (Equation (1)) of three components: exposure, hazard and vulnerability [8,9,10]. The outcome or estimated risk category is related to the combined values of these elements; for instance, a HAB occurrence in a location with an absence of fish (or shellfish) aquaculture farms results in zero overall risk.

*Risk* = *f* (exposure, hazard, vulnerability) (1)

Within Equation (1), the exposure component refers to a subject that could potentially be damaged or lost by the occurrence of a hazard. This definition was utilised in a HAB risk assessment study in Chilean waters, using the averaged fish farm biomass of the last two years as an estimation of the production that could be lost after a HAB event [11].

The hazard refers to a stressor or agent that could cause physical harm or negative effects to a particular activity or in a specific location [7,9,11]. The hazard is related to the event’s frequency, severity and duration, associated with the likelihood of its occurrence. Risk assessment studies have previously included HAB events as hazards to fish farming, amongst other factors beyond the organism’s normal physiological ranges, such as pH, temperature and diseases [11].

The vulnerability component has diverse definitions depending on the question and purpose of the assessment. Vulnerability can be related to socio-economic impacts, including employment levels and economic dependency [12]. This parameter can be used to evaluate susceptibility and the ability to address the hazard, in order to plan prevention and mitigation strategies and build resilience [13]. Vulnerability can be estimated from factors such as exposure to environmental-related impacts, sensitivity of the population to such activity and adaptation capacity of the impacted stakeholders [12,13,14]. However, a direct causal relationship between environmental parameters and HABs is hard to demonstrate [15].

The identification of the main components of risk can allow prevention and mitigation strategies to be developed [4,16,17]. In the last decade, the establishment of monitoring programs, improvement of technology, identification of hazardous phytoplankton species and investigation of HAB physiology have markedly improved [18]. Interest has increased from stakeholders, including governmental, private and public sectors, due to the aquaculture industry’s rapid development, the opportunities this provides to support the income of coastal communities and the potential economic losses associated with HAB events.

Preventative measures such as estimating the carrying capacity of a water body have been important in reducing HAB risk in sea lochs or fjord systems where the water body is located in an enclosed environment [19]. Monitoring of water samples for the identification of harmful phytoplankton and the quantification of biotoxins within shellfish tissue is applied as a measure for safeguarding human and animal health from HAB events [20]. In Scotland, monitoring of phytoplankton that directly impact fish farms is undertaken by the companies themselves, as HAB risk threatens fish health rather than human consumers [21], whilst shellfish-related HAB and toxin monitoring is overseen by the regulatory body Food Standards Scotland. Taxonomic methods for identifying a bloom’s causative species have improved [18], with daily monitoring of water samples and estimation of phytoplankton cell abundance used as an early warning measure by most fish farms [21]. However, the physiological mechanisms that relate harmful phytoplankton to fish health consequences remain poorly understood [5,22] and depend on variables such as fish health and age [23], as well as the multiple factors, including HABs, that generate complex gill disease [24]. There is evidence of a relationship between cell biomass and anomalous fish behaviour, including poor appetite and erratic swimming, such as a study determining a safety threshold of 397 cells/mL of *Alexandrium catenella* in Chile [25]. However, a general cell abundance safety threshold to mitigate HAB impacts on fish physiology has been challenging to determine [26]. A traffic light system is used for ensuring safe shellfish harvesting and posterior commercialisation in Scotland, with standardised monitoring and analysis methods [27]. Fish farm operators use alert levels to assess the risk of HAB events [21], but these vary according to location and company.

**Figure 1:**
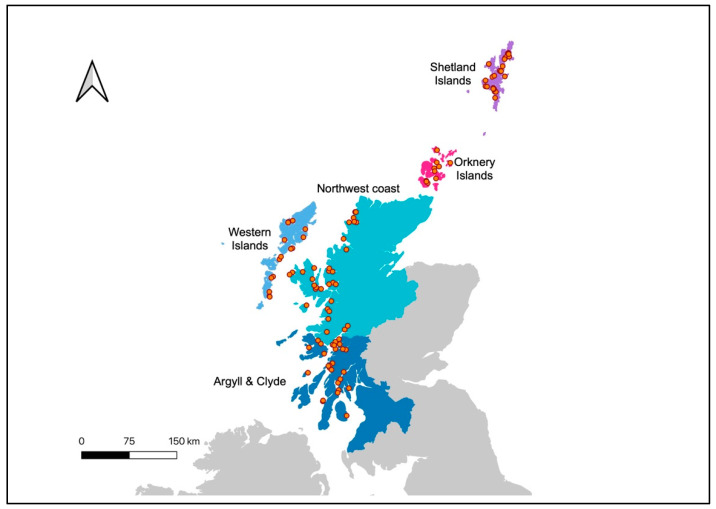
Scottish regions: Argyll & Clyde, the Western Isles, the Northwest coast, the Orkney Islands and the Shetland Islands. Salmon farming sites (orange circles).

Salmon farming is the most important aquaculture resource in Scotland, supporting jobs and livelihoods in coastal areas, with a production of 193,129 tonnes and a value of GBP 932,310,650 in 2020 [28]. The present study synthesised published knowledge of fish farm mortalities associated with HAB occurrence to estimate the regional socio-economic risk of these events to this nationally important industry. Data was available from 2011 to 2020 and broken down within the five geo-political regions of Scotland that undertake salmon farming: Argyll & Clyde, the Western Isles, the Northwest coast, the Orkney Islands and the Shetland Islands (Figure 1).

## 2. Results

### 2.1. Exposure

**Figure 2:**
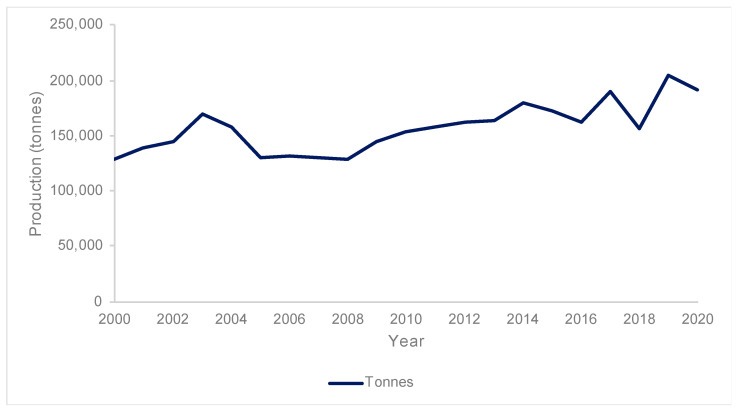
Annual production from 2000 to 2020. Data accessed through Marine Scotland Science [28].

Scottish salmon farming has increased steadily with time over the surveyed period (Figure 2), reaching a maximum production of 203,881 in 2019, with an average of 157,003 tonnes countrywide (2000–2020). On a regional basis, there was significant variation in production (Table 1). The Northwest region accounts for the highest average production, with over a quarter of the country-wide production. The Shetland Islands has the second highest production in the country with nearly a quarter of the total. These are followed by Argyll & Clyde and the Western Isles, whilst the Orkney Islands had the lowest proportion countrywide.

### 2.2. Hazard

The first reports of HAB impacts on farmed fish in Scotland were recorded as ‘flagellate X’, due to the inability to accurately identify these organisms at the time (Table 2). Most likely these blooms were of *Heterosigma akashiwo* (R. Gowen pers. comm.), a species that does not preserve well in the fixatives typically used for microscopy. These events occurred between May and July of 1979 in Loch Striven [29,30] and Loch Fyne in 1982 [29,30,31], both in the Argyll & Clyde region. Later studies reported the occurrence of foam and gelatinous texture associated with *Phaeocystis pouchetti* blooms at fish farm sites in the Argyll & Clyde regions of the Clyde Sea in (1981) [29] and Firth of Forth (a non-salmon-farming region) (~2000) [31], as well as the Shetland Islands (2005) [32]. Although anecdotal reports exist, formally published evidence of diatom species affecting farmed salmon is limited to events in June and July of 1988 in Loch Torridon (Northwest region) and the Shetland Islands. These events were related to the occurrence of Chaetoceros debilis and *C. wighami*, with an estimated economic loss of GBP 4.5 million [33]. The latter species was also associated with fish mortalities (in an unspecified sea loch on the west coast), causing physiological effects such as loss of appetite, lethargic behaviour and loss of 170 tonnes of fish, equivalent to GBP 408,000 [34]. The dinoflagellate *Heterocapsa triqueta* was also reported to be associated with ‘substantial’ fish kills and economic losses in the Shetland Islands in 2001 [35], with a reported 1,000,000 cells/L abundance [32]. Despite these significant losses, blooms of this species are considered rare in Scotland and Norway [30].

The species that has caused a higher frequency of scientific literature-reported fish mortality events in Scottish waters is *Karenia mikimotoi*, previously classified as *Gyrodinium aureolum* or *Gymnodinium* cf. *mikimotoi*. One of the first reports described negative effects related to wild fish mortalities in 1963 in the Moray Firth (northeast coast). This organism was also reported in west coast lochs, with large blooms in Loch Fyne and the Firth of Clyde (Argyll & Clyde region) in September of 1980, with biomass levels of 2000 mg chlorophyll m^−^^3^ in the column water [29,36]. This event was thought to be related to cell advection and the possible presence of cells in the frontal region at the mouth of the Firth of Clyde, leading to the bloom development [36]. This study also suggested thermohaline stratification and freshwater input from land activities could have enhanced cell growth, but this was not statistically explored. Mortalities were associated with hypoxic conditions, cellular gill damage and asphyxiation of 3000 salmon of 1 kg and between 200 and 300 smolts. Other blooms occurred in Argyll & Clyde in Loch Striven, east Loch Tarbert and the Isle of Mull, on the Northwest coast in Loch Ewe [30,36].

*K. mikimotoi* events have been reported in the Northern Isles, such as the Orkney Islands in 1996, causing fish, shellfish and invertebrate mortalities [37]. Several reports identified blooms in the Shetland Islands in 2001 and 2003 [35,38] and a temporally and spatially prolonged bloom that initiated on the southwest coast but eventually reached the Shetland Islands in 2006 [39,40]. This bloom was thoroughly studied due to its large spatial scale and long duration (several months), with one study [41] finding a positive (but non-significant) relationship with irradiance and negative relationships with rainfall and wind intensity. Another study identified rainfall as a significant predictor, which explained a moderate portion (30%) of cell abundance variance [39]. A *K. mikimotoi* bloom was also described in the Firth of Clyde in late July of 2009, causing hypoxic conditions and mass mortality of marine life but no impact on any fish farm since they are absent in this area [42].

**Table 2:** Literature reported phytoplankton bloom events that impacted negatively fish farming in Scotland.

Species	Year	Location	References
Flagellate X	1979	Loch Striven	[29,30]
	1982	Loch Fyne	[29,30,31]
*Phaeocystis pouchetti*	1981	Clyde Sea	[29]
	~2000	Firth of Forth	[31]
	2005	Shetland Islands	[32]
*Chaetoceros debilis*, *C. wighami*, *C. wighami*	1988	Loch Torridon, Shetland Islands	[33]
	1998	Sea loch on the west coast (not specified)	[34]
*Heterocapsa triqueta*	2001	Shetland Islands	[32,35]
*Karenia mikimotoi* (*Gyrodinium aureolum* or *Gymnodinium* cf. *mikimotoi*)	1963	Moray Firth (northeast coast)	[29,43]
	1980	Loch Fyne and Firth of Clyde	[29,36,44]
	1980	Loch Striven, East Loch Tarbert, Loch Ewe	[30,36]
	1982	West and north	[45]
	1996	Orkney and the west coast	[29,37]
	2001	Orkney, Shetland Islands	[32,35]
	2003	Orkney, Shetland Islands	[38]
	2006	Mull (west coast), northwest coast, Orkney Islands, Shetland Islands	[32,39,40]
	2009	Firth of Clyde	[42]
	2016	Loch Ryan	[32]

### 2.3. Vulnerability

**Figure 3:**
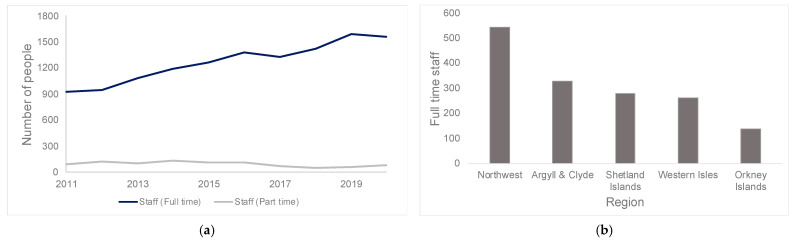
(**a**) Number of staff in full-time and part-time jobs in the Scottish fish farming sector [28]. (**b**) Full-time staff per region (Northwest, Argyll & Clyde, Shetland Islands, Western Isles, Orkney Islands).

The number of full-time staff working in Scottish fish farms has increased since 2011, with part-time employee numbers remaining roughly constant (Figure 3a). Fish farming employed a total of 1557 full time staff and 73 part time staff in the whole country in 2020.

The total number of people employed in the industry varied per region (Figure 3b, Table 3), with the highest number in the Northwest region, followed by Argyll & Clyde, the Shetland Islands, the Western Isles and the Orkney Islands. Calculating the proportion of population that were directly hired, with respect to the total population, allowed us to estimate the dependability on this industry at a regional level. While the Northwest area had the highest number of full- and part-time staff members, it exhibited a relatively low proportion of the population depending on salmon farming. In contrast, in the case of the Shetland Islands, while fish farms employ only the third largest number of people on a regional basis, this region exhibits the highest proportion of the population dependent on salmon farming.

### 2.4. Overall Risk

Risk was estimated using Equation (1) based on the three components explored above, ranging from 1 to 5 for each component and 0 to 125 for the total estimated risk. The Shetland Islands exhibited a ‘very high’ HAB occurrence (hazard) and population dependability on the industry (vulnerability) (Table 4), generally associated with the dinoflagellates *Heterocapsa triqueta* and *Karenia mikimotoi* and the large reported economic losses. The proportion of fish biomass that might be lost (exposure) was ‘high’ in the Shetland Islands, Argyll & Clyde and the Western Isles and ‘very high’ in the Northwest, whilst it was ‘very low’ in the Orkney Islands. The Shetland Islands displayed the highest estimated risk amongst all the regions, with Argyll & Clyde being the second highest. The total estimated risk of the other regions (Western Isles, Northwest and Orkney Islands) were all classified as ‘very low’.

## 3. Discussion

### 3.1. Exposure

Salmon farming production has been increasing worldwide since commercial practices started in the 1980s [47]. This has allowed fish stocking density in Scotland to continue to increase over time, and projections suggest this trend will continue due to increasing food and protein demand [1]. Diverse stakeholders such as risk assessment advisors [16], fish farming companies [23] and the insurance industry [48] perceive HAB events as a major hazard causing fish mortalities, with the severity of incidents potentially increasing in relation to climate change conditions [15,49,50]. Increasing production suggests a greater number of sites will likely be affected in the future.

Salmon is the most important farmed marine species in Scotland, constituting 96% of all production [51]. Increasing biomass, production value and jobs in the last two decades highlight its importance in coastal communities. The greatest production is located in the Northwest area, followed by the Shetland Islands [28]. The spatial extension of these regions differs, with the former ranging from the central west coast to the northwest (~240 km, from limit south to north), while the Shetlands are made up of a group of islands (~110 km, from south to north).

In the present Scottish case study, the production of fish biomass is similar between the five regions (except the Orkney Islands); thus, the decentralisation of salmon production in this country constitutes an advantage with respect to HAB risk.

### 3.2. Hazard

Bloom events have shown heterogeneous temporal and spatial patterns that make their prediction difficult [18,52,53,54,55], associated with diverse causative species between and within countries. HAB reports in the literature mainly focus on events that have caused major economic losses [20], which limits research efforts and understanding of the ecological dynamics that drive other events. This has been confirmed in the present study, where the presence of HAB events in fish farming sites is known [48], but the lack of access to data was a major challenge to the improvement of the risk assessment. Access to recurrent fish mortality data associated with blooms is constrained due to the confidentiality of fish health data held by fish farm companies [56]. This generates difficulties in assessing economic impacts [57] and also prevents easy comparisons at a global or national scale.

Fish mortality associated with HAB events is related to (i) physical or mechanical damage of gill structures [58,59]; (ii) toxic effects from ichthyotoxic species through disruption of osmoregulatory capacity [60], oxidation of cell membranes by reactive oxygen species and polyunsaturated fatty acids [61,62]; (iii) blood hypoxia caused by deoxygenation or decrease of available oxygen in the water column [36,42]; (iv) extreme oxygen supersaturation related to algal photosynthesis, gill lesions and gas bubble trauma symptoms [63,64]. More scientific studies are needed to understand the physiological relationships between phytoplankton and fish health, especially for *Karenia mikimotoi*, which, in this study, has been the most reported HAB species related to fish farms.

In this case study, *Karenia mikimotoi* was reported in the scientific literature to have caused the highest frequency of fish-killing bloom events. This organism was also identified as a key causative species, corresponding to 15% of total global HAEDAT (Harmful Algal Event Database) events recorded [56]. Farmed fish mortalities associated with this species have been ascribed to asphyxiation due to low oxygen levels [36,42], with possible toxicity affecting the gills and other organs [65,66]. Understanding the physiology and dynamics of this species in relation to other variables is limited due to the difficulty of culturing Scottish strains in laboratory conditions. Other strains have been successfully cultivated, with careful considerations in homogenising the culture before sampling due to the high sensitivity of the cells towards agitation of the flasks [67]. A global survey that analysed HAB events recorded in HAEDAT identified *Heterosigma* as a key genus causative of fish mortalities, with major economic impacts registered in Japan, British Columbia, New Zealand and Chile [56]; events in Norway associated with *Prymnesium parvum* occurred in 1988 [68] and 1995 [69]; whilst *Chrysochromulina leadbeateri* has been reported twice over a span of 30 years, in 1989 [6,70] and 2019 [6]. Disease and parasite outbreaks (sea lice, viral and bacterial infections) also play an important role in fish health and, consequently, mortality [71]. Sea lice have also been identified as a major pathogen threat to the development of fish farming [72], with increasing efforts from the industry and academic organizations to mitigate the negative economic impacts these parasites cause.

### 3.3. Vulnerability

Aquaculture practices support livelihoods, employment and economies in coastal communities. Salmon farming employment has increased in parallel with the industry’s development. Assessing vulnerability to a hazard is crucial for understanding socio-economic impacts since HAB events could cause unemployment due to reduced production or closure of sites [73]. This also includes the research and development sector and other indirect jobs in the value chain (transport, supermarkets) or maintenance of the fish farm infrastructure. The present analysis did not incorporate these factors due to the difficulty of accessing relevant data; hence, our analysis may underestimate social impacts.

In the present study, geographically larger regions such as the Northwest coast and Argyll & Clyde reported the lowest full-time worker dependability (proportion of the total population) on salmon farming. Despite the highest number of full- and part-time employment in these areas, the larger populations lower the vulnerability score, implying the availability of alternative jobs during and after HAB events. Islands regions such as the Western Isles and the Orkney Islands exhibited high vulnerability scores related to a low total population. The Shetland Islands presents an exception, as despite exhibiting the highest vulnerability score, it has the lowest unemployment rate across Scotland, with a 2% rate in comparison with 4.8% in Glasgow City or 4.2% in South of Ayrshire (Argyll & Clyde region) [74]; this implies that the community has sufficient employment opportunities to enables workers to change between industries if aquaculture is impacted, in this case by HAB events.

### 3.4. Overall Risk

The estimation of risk as a function of the components of hazard, exposure and the studied locations was useful to assess the socio-economic impacts of HAB events on the aquaculture industry. This function was adapted from other studies since our case study strongly depended on data availability. Future studies should consider a time series of bloom events, extrinsic variables (environmental, climatological, oceanographic) related to their development (especially in the climate change context) and information regarding prevention and mitigation strategies to assess the adaptability capacity [12]. This study also highlights the crucial need to access HAB and fish health monitoring data carried out by fish farming companies to be able to analyse ecological dynamics and quantitatively assess the risk in coastal communities.

The Shetland Islands and Argyll & Clyde scored ‘high’ and ‘medium’ total estimated risk, respectively. These two regions showed the highest proportion of HAB probability (hazard) and proportion of fish biomass produced (exposure) in comparison with the other areas. The Shetland Islands maintained a ‘very high’ vulnerability score, whilst Argyll & Clyde showed a ‘low’ score, potentially associated with the wider spatial range of this region. Although blooms occur mainly in summer months in the Shetland Islands [54], implying high socio-economic impacts, the high employment rate on the island suggests this region’s high resilience towards HAB events due to the availability of alternative jobs.

The other regions, the Western Isles, Northwest and Orkney Islands, presented a ‘very low’ risk, mainly governed by the low hazard score (reported blooms in the literature). A Scottish study that analysed temporal-spatial patterns of HAB species impacting shellfish identified a high variability across the country and within a time series, with both “spring” and “summer” blooms associated with studied locations [54]. The non-significant relationship between fish biomass and HAB abundance, related to the characteristic hydrodynamics and recurrent flushing conditions in the fjords [75], suggests the total cultured salmon is not an important component influencing HAB risk to the Scottish aquaculture industry. This is opposed to Chilean risk assessment vulnerability studies that correlated areas with elevated cultured salmon biomass to high HAB risk occurrence and major negative impacts [12,16]. Differences in the drivers of HAB occurrence, hydrodynamic conditions and stocking density highlight the importance of assessing the risk at a regional or local level since ecological dynamics differ according to geographic locations.

### 3.5. Limitations of the Risk Assessment

Understanding causative conditions related to the development of phytoplankton species that affect fish farms is challenging due to the complexity of their dynamics. The literature reviewed in this study reported and described potential parameters and conditions that could be related to bloom development in Scotland. However, the highly variable temporal and spatial HAB patterns in Scotland [54] constrain the use of environmental variables for risk assessment through a lack of clear correlations. In contrast, risk assessment studies carried out in Chile included environmental variables, such as sea temperature, drought period and eutrophication risk, using modelling projection scenarios in a climate change context [12]. In Chile, eutrophication has been associated with fish farming and has been determined as a serious environmental risk in the Chilean fjords [76]. A study carried out in Chile identified extreme anomalous conditions, including the combined effect of the positive southern annual mode phase and a super-scale El Niño event related to the 2016 HAB event [5]. El Niño events are related to increased sea temperature and drought periods in Chile, which diminishes the freshwater input into the fjords and enhances eutrophication risk [12,77]; thus, these variables were important for assessing HAB risk in this region. The applicability of these variables in Scotland is limited since drought and eutrophication conditions are not often concurring on the Scottish coastline, whilst the climatological driver El Niño is not occurring in the North Atlantic region. The Chilean approach was therefore modified for the present analysis and excluded extrinsic variables due to the difficulty of using these as direct predictors of HABs in the Scottish coastline. However, this highlights the need for an enhanced understanding of the role of environmental and climatological variables in driving HABs and, hence, for estimating future trends and identifying susceptible areas to HAB occurrence.

Adaptation or resilience capacity is a crucial factor in the recovery after exposure to a hazard and in managing its risk, with measures depending on both species and type of aquaculture. Fish farming companies undertake a range of activities, depending on the location and severity of the HAB [21]. Fish farming practices include discontinuation of fish feeding to decrease oxygen consumption and encourage fish to swim in deeper waters that may be below a surface bloom. Relocation of cages to physically avoid a bloom event has also been applied [20] but is rarely practical. Other physical measures such as the deployment of tarpaulins, aeration or development of bubble curtains have been effectively used in some locations in Canada but failed on Chilean and Irish sites [23]. The use of clay as a flocculant agent in Korea has been beneficial to stop the development of the blooms [78]. Modified versions of these clays are now in use [79] but are not yet well-tested outside of Asia. The ability to cope with HAB events is also related to the availability of fish storage for alternative use (i.e., feed), disposal or early harvest. One of the main factors behind the heavy economic loss in the exceptional HAB bloom event in Chile in 2016 was the lack of facilities for fish that were not physically affected [5]. The effectiveness of measures seems to be country- and site-dependent, possibly influenced by the size of the company, water column conditions and HAB species. The importance of the fish farming industry has led to the support and subsidy of a proportion of insurance costs by the Korean government to minimise economic demands on farming practices [20].

Salmon farming companies carry out their own phytoplankton and fish health monitoring [21], but this data is often not publicly disclosed [56]. HAB event data used in the present study was therefore associated with published fish-killing events with major economic losses in the industry [20]. However, we expect that under-reporting of fish mortalities associated with HAB events in Scotland means that these data underestimate the true number of events. More complete records and more robust estimates of vulnerability to HAB occurrences would be valuable not only for fish farming companies but also for the coastal communities that rely on this activity [12]. Going forward, it is intended to establish a standardised phytoplankton monitoring and reporting approach across the Scottish salmon farming industry [21]. A common methodology would enable data sharing using platforms such as HABreports.org portal [80]. The availability of these would allow a better assessment of temporal-spatial patterns and also extrinsic variables driving HAB events.

## 4. Conclusions

This case study presents a preliminary quantification of HAB events risk in the Scottish salmon farming industry. The derivation of a risk function including socio-economic impacts allowed the assessment of this hazard. The highest overall risk evident in the Shetland Islands was associated with the highest score in the hazard, exposure and vulnerability components. In contrast, large geographical areas such as Argyll & Clyde and the Northwest showed a lower total estimated risk and low vulnerability scores, suggesting low dependability on the fish farming industry due to a wide geographical area and diverse job alternatives. This study supports the efforts to identify and quantify the socio-economic impacts of HAB events in coastal communities and highlights the crucial need to access data on phytoplankton (monitoring) and associated fish mortalities to unveil ecological dynamics and physiological consequences to improve the risk assessment of this hazard.

## 5. Materials and Methods

### Risk Assessment Function

HAB risk was estimated as a function of three components: hazard, exposure and vulnerability (function 1). This function was based on case studies that assessed the socio-economic impact of a hazard [8,10,12,13], modified to suit the data available within our Scottish case study.

In our context, exposure was defined as fish biomass production, in this case, salmon (*Salmo salar*), that could be negatively impacted due to HAB occurrence. Data relating to production and its economic value covering from 2000 to 2020 was freely accessed from Marine Scotland Science via the Scottish Fish Farm Production Survey [28]. Exposure per region was estimated as the proportion of farmed fish per region with respect to the total production in Scotland. Hazard was defined as the reported occurrence of bloom events that impacted fish farms in the five study regions in Scotland. Scientific literature was explored and listed chronologically, indicating the species, year and location of the events since the 1960s. The hazard was estimated as the HAB probability of occurrence, using the frequency of blooms reported per region over the total bloom events in the time series (total = 19) from 1963 to 2016. The vulnerability component was used to estimate the socio-economic impact of HAB occurrence on the livelihoods of those working in Scottish fish farms. Data on full-time and part-time employees was accessed per region from the Scottish fish farm production survey [28]. This was converted to the proportion of people working in fish farms with respect to the total population in the region, using freely accessed data demographics from the Mid-2020 population census from the National Records of Scotland [46]. Salmon farms’ prevention and mitigation approaches after a HAB event vary between sites and companies and are not detailed in the published literature; hence, these were excluded from the vulnerability component of our analysis.

A simple scoring system was used to assess risk per region; components ranged from 1 to 5, each carrying an equal weight (Table 5). The maximum probability of each component per region was calculated, then the integral rounded number (Hazard = 0–40%; Exposure = 0–30%; Vulnerability = 0–1.3%) was divided in five quantiles and classified from 1–5 in numerical order. The maximum being the ‘very high’ category, followed by ‘high’, ‘medium’, ‘low’ and ‘very low’. This approach was modified from [12], who assessed diverse threats (water temperature, salinity, HAB occurrence, amongst others) impacting salmon farming in Chile. The score values (1–5) were then multiplied using the function of risk (descending order in Table 5); thus, the maximum estimated risk is 125 (5 × 5 × 5).

## Figures and Tables

**Table 1:** Average production (tonnes) and value (GBP) from 2011 to 2020 per region [28].

Region	Annual Production (Tonnes)	Production by Value (GBP)	Regional Proportion Produced (%)
Northwest	49,053	238,024,903	28.21
Shetland Islands	39,356	190,085,645	22.63
Argyll & Clyde	36,417	179,424,386	20.94
Western Isles	34,504	168,395,387	19.84
Orkney Islands	14,548	72,870,687	8.37

**Table 3:** Direct employment (full-time = F/T staff), part-time employment (P/T), total population per region, proportion of the population depending on salmon farming (%) (Data collected in 2022 by Marine Scotland Science [28]).

Region	Full-Time Staff	Part-Time Staff	Total Population	Population Dependent on Salmon Farming (%)
Shetland Islands	280	12	22,870	1.22
Western Isles	262	12	26,500	0.99
Orkney Islands	138	13	22,400	0.62
Argyll & Clyde	331	17	85,430	0.39
Northwest	546	19	235,430	0.23

**Table 4:** Estimated risk per area, using components Hazard (HAB probability, calculated as the frequency of blooms per region over the total time series (n = 19)), Exposure (fish produced per region in relation to country production) and Vulnerability (population depending on fish farms over the total Scottish population [46]). Score values were assigned depending on maximum probability values and quantile classification (described in methods), included in the estimated risk function Risk = f(HxExV).

Region	Hazard		Exposure		Vulnerability		Estimated Risk R = f(HxExV)
	HAB Probability (%)	Score	Proportion Produced (%)	Score	Population Depending on Fish Farming (%)	Score	
Shetland Islands	33.33	5	22.63	4	1.22	5	100
Argyll & Clyde	38.89	5	20.94	4	0.39	2	40
Western Isles	5.56	1	19.84	4	0.99	4	16
Northwest	16.67	2	28.21	5	0.23	1	10
Orkney Islands	22.22	3	8.37	1	0.62	3	9

**Table 5:** Risk categories are specified per component score and estimated risk. Scores per component (‘Hazard’, ‘Exposure’, ‘Vulnerability’) were assigned 1 to 5, using the maximum probability divided into five quantiles. The total estimated risk outcome is the product of the multiplication of components: hazard, exposure and vulnerability. The maximum estimated risk was calculated as the product of maximum scores (5 × 5 × 5 = 125), with the range hence being from 0–125.

Risk Category	Score (1–5)	Hazard (0–40%)	Exposure (0–30%)	Vulnerability (0–1.30%)	Total Estimated Risk (0–125)
Very-high	5	33–40	25–30	1.10–1.30	101–125
High	4	25–32	19–24	0.76–1.00	76–100
Medium	3	17–24	13–18	0.51–0.75	51–75
Low	2	9–16	7–12	0.26–0.50	26–50
Very-low	1	0–8	0–6	0–0.25	0–25

## Data Availability

The data supporting the research of this article will be made available by the authors, without undue reservation. The total fish biomass, number of full- and part-time jobs in the Scottish fish farming sector can be accessed through the updated annual reports published by Marine Scotland Science; in this study, we used the latest (2021). The demographics information per region was accessed through the National Records of Scotland, published in 2021.
